# On Some of the Causes of Puerperal Convulsions

**Published:** 1858-10

**Authors:** Ralph N. Isham

**Affiliations:** Chicago, Ill.


THE CHICAGO

### MEDICAL JOURNAL.

VOL. I. OCTOBER, 1858. No. 10.

### ORIGINAL COMMUNICATIONS.

ARTICLE I.

### ON SOME OF THE CAUSES OF PUERPERAL CONVULSIONS.

BY RALPH N. ISHAM, M.D., CHICAGO, ILL.

The growing interest of the profession in this subject, the research which has been made and is going on in the field of organic chemistry, in its relations to eclampsia, resulting in the exposition of facts so striking and important to us as practitioners, for study and reflection; not only excuse but seem to invite even the humblest efforts to fix our thoughts upon this terrible complication of childbirth, which brings dismay to all who witness it.

It may not be amiss, even in this short paper, to more particularly define my subject than is meant by the generic term eclampsia, and I quote for that purpose the following extracts:*

"Eclampsia puerperalis is an acute affection of the motor function of the nervous system, characterized by insensibility, tonic and clonic spasms, and occurs only as an accessory

* Uremic Convulsions of Pregnancy, etc., by Dr. Carl R. Braun, Prof., etc.: Translated by J. Matthews Duncan. Edinburgh, 1857.

phenomenon of another disease, generally Bright's disease, in an acute form, which, under certain circumstances, spreading its toxæmic effects on the nutrition of the brain and whole nervous system, produces those fearful accidents." The toxæmia in the convulsions of pregnancy, parturition and childbed, is commonly produced by uræmia, *i. e.*, by a change of the urea which has been retained in the blood, or by retention of excrementitial extractive matter of the urine.

The same disease with similar phenomena may manifest itself in women who are not pregnant, in children, and even in males under certain circumstances favorable to it; yet by true puerperal eclampsia, is now implied the symptoms during pregnancy or childbirth intimately connected with diabetes albuminosus.

"It is distinguished by quick repetition of the fits, and complete insensibility during the fit, as well as generally during the interval. The face and neck appear swollen and injected during a paroxysm; the eyelids are prominent, and open or closed; the eyeballs exhibit quick rolling motions in the most different directions, or are fixed in an upward stare; the vessels of the conjunctiva are mostly injected; the mouth is at first widely opened and distorted; the tongue is protruded; then trismus follows, in which, if proper care be not taken, the protruded tongue is often bitten through, and hence a bloody foam often flows out of the mouth."

"In the muscles of the face lively distorting convulsions are observed, whereupon the upper extremities get bent, the trunk is twisted to one side, and then all the extremities are thrown into jerking motions. Respiration often altogether ceases for many seconds; the carotids show strong pulsations; the veins of the face and neck swell on account of stoppage of the blood from muscular spasms; the color of the face cyanotic. All the muscles of respiration, especially the diaphragm, are in a state of contraction, and in consequence of this, asphyxia may occur. The urine and fæces are involuntarily excreted. Vomiting rarely precedes the first fit. The skin remains dry, or may be covered with perspiration, and its temperature is either increased or diminished; the reflex sensibility is suspended during the fit; the pulse is frequent or slow; the arteries large or small. After this group of symptoms, there follows a soporose condition, in which the patient continues for a shorter or longer time, and lies motionless; the extremities stretched out and stiff; the respiration frequent and difficult, and at first stertorous, afterwards slower and snoring. Generally there is absence of consciousness and sensation.

"The duration of each fit, including the tonico-clonic part, and the soporose part, extends commonly to half an hour, or to one or two hours, and only in very rare cases does the sopor of the first fit last a whole day.

"If the paroxysm does not terminate in death, a remission takes place; the respiration becomes more slow and free and less rattling; the rigidity of the muscles diminishes; the frequency of the pulse becomes considerably less; consciousness either does not return at all or only very slightly, mostly remaining dim, so that a proper but short answer may be got to a question in a loud voice, but recollection of what has happened is altogether wanting; the abdomen is sensitive to touch, and the reflex sensibility is often intense during this lighter sopor. After awaking, patients generally complain of a confined, dull headache, and of great languor, which continue till a renewal of restlessness, stretching, extending, slow, tremulous bending of the upper extremities, jerking of the facial muscles, with reddening of the face, announce a new paroxysm. The fits may be repeated several times in a day, sometimes as much as seventy times. Generally, after a few fits, complete unconsciousness supervenes, and this continues till recovery or death." *

There is little chance of confounding this variety with the common hysterical form which is frequent in the early months of pregnancy, or those of epilepsy, catalepsy, or chorea. Cerebral or apoplectic eclampsia is described as generally originating in the rupture of some cerebral vessel, or in hyperæmia of the brain, which may, under some circumstances, closely resemble an eclamptic attack; but in the latter, the spasms continue and occur in the paralyzed parts; there are no intervals of consciousness; the profound coma precedes the convulsions instead of

* Braun—*op. cit.*

following them as in uræmic eclampsia; the breathing is slower and quieter; the pulse slow and hard; the symptoms of disease of the kidneys are absent, and are either followed by a paralysis of the opposite side of the body from that of the brain, which is the seat of disease, or by idiocy or death. Without doubt, cases of uræmic convulsions may terminate as apoplectic by fatal effusions upon the brain, as indicated by post-mortem appearances. This is easily understood when we reflect upon the great congestion which is present in cases where there is a predisposition, as regards habit, etc. And such cases would be difficult to define accurately.

*Relations of Albuminuria to Puerperal Eclampsia.*—Fifteen years ago, Dr. John C. W. Lever called the attention of the profession to the important fact, that in puerperal convulsions the urine is proved to be albuminous; since that time, by more careful analysis of cases and accumulated evidence, through the observations of Simpson, Dubois, Cazeaux, Blot, Frerichs and many others, the coincidence of the albuminous urine with eclampsia is now fully established and undisputed, and no well observed cases show this state of the urine to have been absent. In over fifty per cent of fatal cases of convulsions when autopsies have been made, undoubted Bright's disease has been found, which has led to a series of investigations by Frerichs, Braun, Litzmann and others, who strenuously maintain the *identity* of uræmic intoxication in acute Bright's disease and puerperal eclampsia; amongst the proofs of which are the following:

1*st.* The same precursory symptoms, of which œdema is the most frequent.

2*d.* The same renal lesions are found after death in eclampsia and Bright's disease.

3*d.* The identity of the convulsions, and the fact that males may be subject to them in morbus Brightii.*

4*th.* The two diseases are always accompanied with albuminuria.

5*th.* Bright's disease, as well as eclampsia, sometimes exists without the presence of œdema.

---

* M. Lenoit—*Moniteur des Hopiteaux*, 1857.

In forty-one cases of albuminuric women, Blot met only seven cases of eclampsia; in the same way, Bright's disease is only accompanied by convulsive affections in some cases. Not only has fifty per cent of the autopsies revealed Bright's disease, but in negative observations the histology of the kidneys was microscopically examined only in rare instances. The remarkable agreement as to gravity and termination between eclampsia and Bright's disease is worthy of notice.

A recent writer, M. Goubeyre, in a prize essay read before the Paris Academy of Medicine, asserts that there is a puerperal Bright's disease just as there is a puerperal peritonitis or puerperal pneumonia, and that the convulsion is a symptom of the disease, or that puerperal albuminuria is neither more nor less than Bright's disease, but is at issue with Frerichs and those who explain the cerebro-spinal complications so often met with in Bright's disease by the theory of uræmic intoxication, not having been able to confirm their observations of the *presence of ammonia in the expired air.*

On the contrary, a strong array of talent attempts to show that the degeneration of the kidneys which has been found in the examinations of those who have died of eclampsia, is the result of the convulsions merely, produced by hyperæmias or serous congestions, being purely accidental secondary phenomena, and it is urged:

1*st.* That persons dying of eclampsia have shown only in a majority of cases (not all) a condition of kidney justifying a diagnosis of Bright's disease.

2*d.* That eclampsia is sometimes developed by fear.

3*d.* That eclampsia has also in some women appeared in successive pregnancies, and that this fact cannot be reconciled to the theory of its being Bright's disease.

4*th.* That it is not proved that albuminous urine always precedes the outbreak of convulsions, but, on the contrary, in many cases this condition of urine is produced for the first time during the delivery or convulsions.*

The first objection is quite a sufficient one to the ground

* Scanzoni.

which assumes that the identity between Bright's kidney and eclampsia is complete, if (a fact, shown by a case which follows) any cases of eclampsia do occur when the Brightian degeneration of the kidneys is proved not to have been present, and we must look elsewhere for the solution of those cases. To the second objection scarce any importance should be attached, for there is not sufficient evidence by accurate knowledge that such were cases of *true* eclampsia falling under the definition heretofore given.

To the third objection, it is only good against the arguments of those who, like Goubeyere, maintain the identity of *acute* Bright's disease and eclampsia. It is not a valid objection to the theory of uræmic intoxication as a cause; for why should not this condition be produced in chronic organic degeneration of the kidneys when disalbumenization is going on, aggravated by the condition of pregnancy and increased circulation? Lumpe has published a case in which eclampsia occurred in the first, second and fifth deliveries. Three hours after the last, death took place, and on a dissection being made, there was demonstrated beyond doubt, in the left kidney, the second stage, that of fatty degeneration; and in the right kidney, the third stage, that of atrophy of Brightian kidneys. In order to call attention to another condition of the kidney, which has been greatly underrated in importance and overlooked, I quote the following case, recorded by my tutor in obstetrics: *

"Case Sixth.—Emily Gray, an unmarried Irish woman, thirty years of age, was driven from her home in the seventh month of her pregnancy, and came to this country without friends or money.

"Nov. 21st, 1852, at 8 p.m.—Her labor commenced under the care of Mr. Peck, Mr. Walker and Dr. Mizener, at 1½ a.m. The os was fully dilated and the membranes ruptured, and at 2 a.m. she was in convulsions. Between the third and fourth convulsion, chloroform was exhibited by Dr. M., and I arrived at the house just at the termination of the fourth. No other

* Case of Albuminuria, in relation to Puerperal Convulsions. By Geo. T. Elliot, jr., M.D , one of the Physicians to Bellevue Hospital.—*New York Medical Times*, July, 1853.

treatment had been resorted to. I found the pulse frequent, feeble and compressible; utter unconsciousness, with stertorous respiration; eyes partially open, and pupils somewhat dilated; no œdema of the feet or legs, or marked puffiness of the face; bladder somewhat distended, and when relieved by the catheter, nitric acid showed the urine to be loaded with albumen; parts well dilated; head presenting in the first position, and well down; pains moderate, and fœtal heart inaudible. At the commencement of the fifth convulsion, chloroform was exhibited, and its use continued until I had extracted a dead child with the forceps, removed the afterbirth and applied the binder. The uterus remained well contracted, and she had no other convulsion; her pulse was feeble, but rallied, and she was ordered an injection of soft soap and salt; gr. xij. of calomel were given in butter, and Granville's lotion applied to the nape of the neck. Consciousness returned in about two hours.

"1 p. m.—Found her complaining of fixed pain in the top of her head, and some reaction commencing; bladder relieved by catheter; ℥ij. of blood were taken by cups from the temples; cream of tartar as a drink, with ʒij. of sweet spirits of nitre during the day; feet and legs kept warm; absolute rest enjoined. In the evening she was restless, and the gentlemen gave her a full anodyne.

"Nov. 29th, 1 p. m.—Much improved; pain in head gone; pulse good; mind anxious and desponding; complains greatly of her tongue, which was severely bitten; decided pain on pressure over her kidneys. Ordered to gargle her mouth with a weak solution of chloride of soda; saline drinks to be continued; bowels to be moved with an injection; and cups to be applied over the lumbar region.

"Nov. 30th, 1 p. m.—I found her extended *on the floor*, apparently dead. I revived her, and lifted her into bed, when I learned that, desiring to have a motion, she had arisen three times, and that on succeeding she had fainted. I impressed on her, and on the kind old woman who had afforded her a shelter in her solitary little room, that such another imprudence would be probably fatal; and as the pulse was returning to its usual state, I left, after requesting that the catheter might be passed, as the bladder had not been emptied since the night before. She objected to its use; and the gentlemen left, promising to return. When they did so, the nurse pronounced her asleep, and they found her dead. She had been again in the upright position, and had had some of her linen changed, just after which she had sunk on the bed, asleep as the old nurse thought. I would especially mention, that she had passed a very comfortable night, and had expressed herself as much better on the morning of her death. Not more than ℥vj. of blood had been taken by the cups.

"*Autopsy.*—Twenty hours after death, by my friend, Doctor C. E. Isaacs. Brain firm and healthy, but remarkably pale—even the choroid plexus being of a much lighter color than natural; all the other organs healthy, saving the kidneys, which were found to be enlarged and congested, *but not changed in structure;* the corpus luteum was beautifully marked; the urine which I drew off with the catheter before delivery was examined by Prof. Alonzo Clark, and found to contain blood corpuscles, *but no casts or fat globules.*

"I may mention that although the feet and legs were not swelled when I was called to see her, she had complained of very great inconvenience from that cause up to within a short time of her confinement, affording an evidence of the fallacy of deducing the non-existence of albuminuria from the absence of this symptom.

"She had resorted to very tight lacing as a means of concealing her pregnancy, and I have (says the Doctor) in my possession the iron corset-bone which she used to assist her."

In this case, recorded as one of true eclampsia by Dr. Elliot, it will be seen that there was no Bright's disease, so pronounced by the highest authority; but in the tight lacing which this patient resorted to in order to conceal her pregnancy we have, I think, a solution of the case. How abundantly does it prove that the pressure of the gravid uterus, assisted by the efforts of the patient by means of dress (more generally in refined life to improve the figure), prevents the proper circulation of the blood through the kidneys, by pressure upon the renal veins, or the continued pressure upon the inferior vena cava, by which the renal capillaries are dilated through the increased hydrostatic pressure of the blood, consequently followed by congestion and an albuminous condition of the urine, produced by a paralysis of the bladder, a symptom which I attribute in this case to the effects of pressure.

A case is recorded by Picard,* of a male patient with stricture of the urethra, who died of albuminuria and *eclampsia*, and on dissection, *acute* Bright's disease was found, which had undoubtedly originated in congestion; indeed, cases are familiar to almost every surgeon, where stricture of the urethra, or paralysis, giving rise to retention, has produced convulsions and coma from the toxæmia, by decomposed urine and the resorption of ammonia; the presence of which is often strongly indicated by the odor of the urine and its iridescent appearance only.

Retention of urine from morbid irritability of the neck of the bladder by the gravid uterus, is not a very rare accident. Braun states a case of retroversion of the uterus, which ended fatally under eclamptic attacks, in consequence of Bright's degeneration of the kidneys and secondary uræmia.†

In the latter months of pregnancy, the uterus exerts, under the same circumstances, the same pressure upon the fundus of the bladder, producing a paralysis which is oftentimes not relieved until long after delivery; retention of urine, *per se*, if neglected, it is well known, will produce a retroversion of the uterus, and whilst serious injury may befall the bladder, greater mischief is produced by the interference in the circulation and the congestion produced. Retention in these cases is seldom complete, and through the modesty of the patient, the physician is seldom applied to for relief until the disease assumes a graver aspect.

The sympathy between the bladder and kidneys is well understood, that a slight irritation of the neck of the bladder will produce a great change in the renal secretions, whilst the changed condition of the latter exert injurious effects upon the

---

* Picard—*Gazette de Strasbourg*, No. 7, 1855.

† Dr. Carl Braun—*Obs. Clinique.*

integrity of the former. For the urine to be excreted normally by the kidney its free expulsion must be secured, and when distention of the bladder happens, the pelvis and ureters become similarly affected, and they also employ active and injurious efforts to obtain the propulsion; hence the congestion and subsequent albuminuria.

Civiale states that he has met with cases of albuminuria and diabetes insipidus dependent upon neuralgia of the neck and consequent atony of the body of the bladder. In these cases, disease of the kidney proves the immediate cause of death, as indeed in the majority of the affections of the urinary apparatus. It is generally conceded that tumors so situated as to press upon the emulgent veins or ureters, have been productive of interruption of the eliminative action of the kidneys. Sir Benj. Brodie says that a tumor pressing upon *one* ureter will sometimes stop secretion of urine from *both* kidneys; terminating, sometimes, in death by coma, accompanied with convulsions. The following is the synopsis of a case which occurred under my own observation, as one of the House Physicians of Bellevue Hospital, in January, 1855, which will illustrate to a certain extent the truth of these accidents:

The patient, A. G., a robust, sanguineous primipara, was seized with pains at 4 p. m., February 13th. The pains of the second stage were very severe, and the first convulsion occurred with the birth of the head. She had complained of no cephalalgia during labor, no nausea, nor perversions of vision or hearing; bowels had moved regularly, and twice moved previous to commencement of labor. The convulsions commenced with grating of the teeth, traction of head to left side, rolling of eyes, convulsive movements affecting the upper portion of the frame, and gradually invading the whole body, especially the left side. Face became livid and swollen; eyes rolled upwards, and sharp nictation of lids, pupils alternately dilated and contracted; the mouth closed and distorted; lips livid and covered with a bloody foam; legs rigid and straight; arms drawn up. The first attack lasted about one minute, leaving her natural both in appearance and manner, but as the convulsions more frequently occurred, she became more profoundly comatose, recovering less perfectly during the intervals. The progress of the labor was uninterrupted. Number of convulsions, eighteen; duration of labor, twenty-two hours. Child asphyxiated, but restored by artificial respiration. Patient died half an hour after delivery, comatose.

*Autopsy.*—Eight hours after death. Brain healthy but congested; lungs œdematous; heart natural. The uterus was large, weighed two and one quarter lbs. avoirdupois, extending above the umbilicus, and pushed to the right side; the right ureter and the pelvis of the right kidney were enormously distended by urine; the bladder was filled but not distended; no abnormal appearance of the left urinary apparatus; the kidneys were small, lobular and fatty, under the microscope. Sp. gr. of the urine 1020, highly albuminous; fourchette not fully lacerated. In this case, there was impediment to the circulation of the right kidney, whether produced by the distension of the pelvis of the kidney and its ureter, or the retardation of the venous circulation in it, by the pressure of the gravid uterus, we cannot say with any certainty; and I regret that my notes of the case are not exact enough to state the difference in the pathology of the kidneys, yet we are to regard it as the immediate cause of the attack of convulsions, whilst the organic change was a predisposing cause, and I think we are not to suppose that had the free discharge of urine occurred, and the circulation been unimpeded, such an accident would not have taken place. There were no premonitory symptoms in this case to indicate the approach of the attack, and it will be seen that it was one of *tedious* labor, the duration of which was twenty-two hours, and the unequal pressure may not have taken place until the onset of the labor, when the partial or entire stoppage of the eliminative process of the kidneys (already diseased) gave rise to the sudden uræmia.

This may be considered as a type of those cases which sometimes occur without any œdema of the extremities, eyelids, etc., or head symptoms. The probability that convulsions may be induced thus suddenly by the congestion produced in a case of tedious labor, is heightened, when we consider that the elimination of urea from the blood by the kidneys is so rapid that under ordinary circumstances it is never allowed to exceed one-fiftieth of one per cent. of the circulating blood. Dr. Shearman has recorded the case of a boy, aged eight years, who was run over by a truck which passed over his loins, evidently inflicting some severe internal injury. From the collapse which at first supervened, he recovered, under the influence of warmth and stimulants, but he passed no urine for thirty-six hours after the accident, and that which was then discharged contained blood. Dr. Shearman carefully examined this urine, but failed to detect the least particle of urea or urates in it. Sixty hours after the accident there was considerable access of fever, with increasing pain in the region of the kidneys, and these symptoms were succeeded by coma. The boy was bled from the arm; and on making a chemical examination of the blood, urea was most distinctly detected in it, and in considerable quantities; the urine, at the same time, not containing a particle of urea, urates or uric acid, and its sp. gr. being only 1005.* Although the presence of urea in excess in the circulation will produce coma and death, yet not to this cause only are we to attribute the uræmic fits. Urea injected into the veins of animals has been tolerated without any evil consequences.†

Frerichs has been led, by a series of experiments, to the following conclusions, which have been confirmed by Braun:

The phenomena of uræmic eclampsia are not produced by urea or other excretory matters of the urine alone, but from urea accumulated in the blood, and converted by some unknown process into carbonate of ammonia; and that to the presence of the latter in the circulation is the eclampsia owing.

In persons who have died of Bright's disease, the blood has been found to contain urea in quantities, without any consequences attributable to the fact during life; hence it is supposed from that fact that the peculiar ferment was wanting to convert the urea into carbonate of ammonia. The cause of this change is as yet not known.

TO BE CONTINUED.

---

* *Edinburgh Monthly Journal*, March, 1858.
† Johnston on Disease of Kidneys.

